# Spotted Fever.—Committee of the American Medical Association

**Published:** 1864-12

**Authors:** 


### SPOTTED FEVER.—COMMITTEE OF THE AMERICAN MEDICAL ASSOCIATION.

The Committee of the American Medical Association to which was referred at its late annual meeting the subject of "*Spotted Fever—so called*" earnestly solicits from members of the Profession in different parts of the country information respecting the history, phenomena and treatment of this disease as it has come under their own notice.

As it is desirable to know the geographical distribution of this disease, intelligence respecting it from physicians residing in distant States, and from Medical Officers of the Army is particularly requested.

For the sake of uniformity the following questions have been prepared. Any matters not included in these questions or suggested by them, will, with the answers themselves be gratefully appreciated and acknowledged by the Committee,

Address the Chairman of the Committee

Dr. James T. Levick,
1109 Arch Street, Philadelphia.

1. When did "*Spotted Fever—so called*" appear in your neighborhood, and how long did it prevail there?
2. What were the usual symptoms of the disease, and what unusual symptoms occurred in your practice?
3. Did it attack many individuals at the same time, and was it materially modified by the age, sex or temperament of the patient?
4. What was the ordinary duration of the disease? Were relapses or severe attaks common?
5. Are you in possession of any proof that the disease was communicated from one person to another?
6. What appeared to be the predisposing and what the exciting causes of the disease?
7. What complications and what sequelæ of this disease came under your notice?
8. What other disease prevailed at or near the time that Spotted Fever did, and what epidemic diseases followed it?
9. What was your mode of treating this disease?
10. What was the proportion of deaths to the whole number of persons attacked, and what was the usual manner of fatal termination?
11. What were the *post mortem* appearances?
12. Were any microscopical observations made, and what were they?
13. Has this disease prevailed in your neighborhood in former years.

